# *MUC4*, *MUC16*, and *TTN* genes mutation correlated with prognosis, and predicted tumor mutation burden and immunotherapy efficacy in gastric cancer and pan‐cancer

**DOI:** 10.1002/ctm2.155

**Published:** 2020-08-22

**Authors:** Yue Yang, Jieyun Zhang, Yanxing Chen, Ruihua Xu, Qi Zhao, Weijian Guo

**Affiliations:** ^1^ Department of Medical Oncology Fudan University Shanghai Cancer Center Shanghai P.R. China; ^2^ Department of Oncology Shanghai Medical College Fudan University Shanghai P.R. China; ^3^ Department of Medical Oncology State Key Laboratory of Oncology in South China Collaborative Innovation Center for Cancer Medicine Sun Yat‐sen University Cancer Center Guangzhou P.R. China

Dear Editor,

Previous studies reported that *MUC16* mutation was associated with better prognosis and higher tumor mutation burden (TMB) in gastric cancer, while *TTN* mutation was associated with better response to immune checkpoint blockage in solid tumors, but the potential mechanisms were still unclear.^1^, ^2^ Through the analysis in TCGA gastric adenocarcinoma cohort (N = 443) and FUSCC gastric cancer cohort (N = 177), we identified two mucin genes, *MUC4* and *MUC16*. These two mucin genes were selected based on mutational frequencies in gastric cancer, gene length, correlation with prognosis, and previous studies (Table S1, Figure S1). We further included the longest gene, *TTN*, into analysis, in consideration of its high mutation frequency and close correlation with TMB.

We observed high potency *MUC4*, *MUC16*, and *TTN* had in predicting TMB in both TCGA and FUSCC cohort. *MUC4*‐, *MUC16*‐, and *TTN*‐mutated cancer showed higher TMB (Figure 1). Mutation numbers of *MUC4*, *MUC16*, and *TTN* were closely correlated with TMB (Table S2). In TCGA cohort, correlation coefficient reached the highest of 0.782 when combined mutation numbers of three genes together; while in FUSCC cohort, the correlation coefficient reached the highest of 0.748 for *MUC16* plus *TTN*, and was 0.735 for three genes. The receiver operating characteristic (ROC) curve further proved the efficacy using mutation numbers to predict TMB (Figure 1, TMB high was defined as top 20% in each cohort, TCGA: >20 mutations/Mb, FUSCC: >8 mutations/Mb). In TCGA cohort, area under ROC curve (AUROC) reached the highest of 0.936 when combined three genes together; while in FUSCC cohort, AUROC reached the highest of 0.925 for *MUC16* plus *TTN*, and was 0.915 for three genes together. The Youden index is shown in Tables S3 and S4. In both TCGA and FUSCC cohort, high TMB was correlated with better overall survival (OS) (Figure 1). High mutation number of *MUC4*, *MUC16*, and *TTN* was correlated with better OS in TCGA cohort, while showing a trend of better OS in FUSCC cohort (Figure 1, high mutation number was defined as top 20% in each cohort).

**FIGURE 1:**
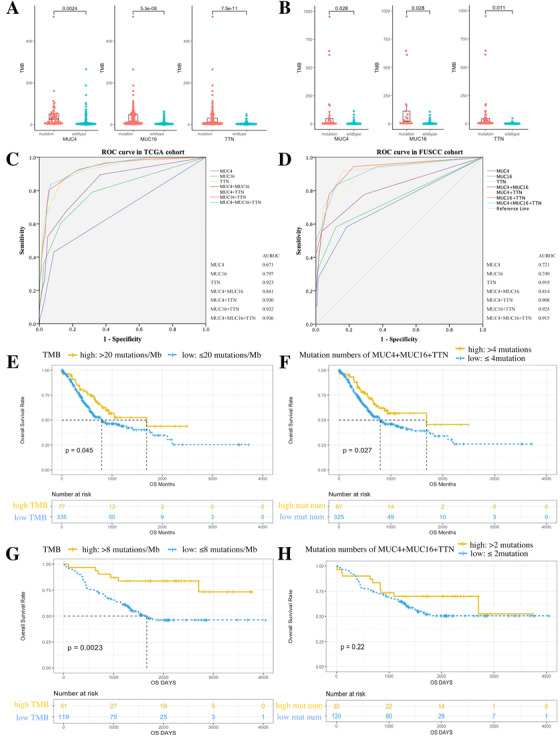
Mutation status and mutation number of *MUC4*, *MUC16*, and *TTN* predict TMB and prognosis in TCGA and FUSCC cohort. A and B, *MUC4*, *MUC16*, and *TTN* mutations showed higher TMB in TCGA and FUSCC cohort. C and D, ROC curve using gene mutation numbers to estimate TMB status in TCGA and FUSCC cohort. E and F, Kaplan‐Meier survival analysis stratified by high‐TMB and high mutation numbers in TCGA cohort. G and H, Kaplan‐Meier survival analysis stratified by high‐TMB and high mutation numbers in FUSCC cohort

In order to realize whether mutation of *MUC4*, *MUC16*, and *TTN* gained functional change or just contributed to TMB, we further analyzed the gene mutation sites distribution (Figure S2).^3^, ^4^ Somatic mutations of *MUC16* and *TTN* were sporadic in both TCGA and FUSCC cohort. Only *MUC4* gene had a slightly high mutation rate of H4205Q in TCGA cohort (2.04%) and V3305_S3320del in FUSCC cohort (7.34%). The univariate and multivariate survival analysis showed H4205Q mutation was independently correlated with worse OS in TCGA cohort (Table S5, HR (95% CI): 2.266 (1.028‐4.994), *P* = .043), while V3305_S3320del was independently correlated with better OS in FUSCC cohort (Table S6, HR (95% CI): 0.221 (0.053‐0.928), *P* = .039). Of note, TMB and mutation numbers were not independent prognostic factors, which indicated them as marker for prognosis but not determining factors.

We further clarified the potential mechanism why *MUC4* and *MUC16* mutation, high TMB and high mutation numbers were correlated with prognosis. In both TCGA and FUSCC cohort, patients with MUC4 mutation showed lower T stage, while *MUC16*‐mutated patients showed lower N stage. High TMB and high mutation numbers were correlated with lower N stage in TCGA cohort but lower T stage in FUSCC cohort (Table S7, *P* < .05). In both cohorts, *MUC4*, *MUC16*, and *TTN* mutation showed alternations in cell signaling pathway, immune checkpoint expression, and immune cell infiltration (Figures S3–S6). High TMB cancers upregulated myc, cell cycle, metabolism, and DNA repair pathways; at the same time, they also showed upregulation of immune response pathway and high infiltration of CD8 T cells, CD4 T cells, macrophage 1, and macrophage 2 cells (Figure S7). These findings indicated that high TMB was accompanied with high genetic instability. Under this circumstance, oncogenes and metabolism genes had more opportunity generating mutations, but also more neoantigens were produced to stimulate immune response.^5^ So the prognosis might be based on a comprehensive consideration of disease status and treatment.^6^ High mutation numbers showed similar results on cell signaling pathways, immune cell infiltration, and immune checkpoint expression to TMB, indicating that gene mutation number might be the maker of TMB (Figure S7).

We verified our hypothesis in one gastric cancer immunotherapy dataset from Sun Yat‐sen University Cancer Center (SYSUCC cohort)^7^ and another pan‐cancer immunotherapy dataset from one published study.^8^ In both SYSUCC and pan‐cancer cohort, mutation status and mutation numbers of *MUC4*, *MUC16*, and TTN were closely correlated with TMB and showed high potency in predicting TMB (Figure 2; Tables S2, S8, and S9). In SYSUCC, although single gene mutation showed limited association with objective response rate (ORR), OS, and progression‐free survival (PFS) (Table S10, Figure S8), mutation numbers of combined *MUC4*, *MUC16*, and *TTN* showed similar efficacy with TMB in predicting immunotherapy effects, as similar in ORR, weaker in OS, but better in PFS than TMB (Figure 2, mutation numbers vs TMB, ORR: *P* = .036 vs *P* = .017; OS: *P* = .17 vs *P* = .038; PFS: *P* = .016 vs *P* = .055).^7^ In the pan‐cancer dataset, *MUC4* mutation presented limited association with OS, while *MUC16* and *TTN* mutations showed significantly better OS, respectively (Figure S9). Mutation numbers of combined *MUC4*, *MUC16*, and *TTN* was more powerful than TMB in predicting OS, while none of them met statistical significance in PFS or ORR (Figure 2, Table S11, mutation numbers vs TMB: OS: *P* = .002 vs. *P* = .041; PFS: *P* = .56 vs *P* = .49; ORR: *P* = .59 vs *P* = .064).

**FIGURE 2:**
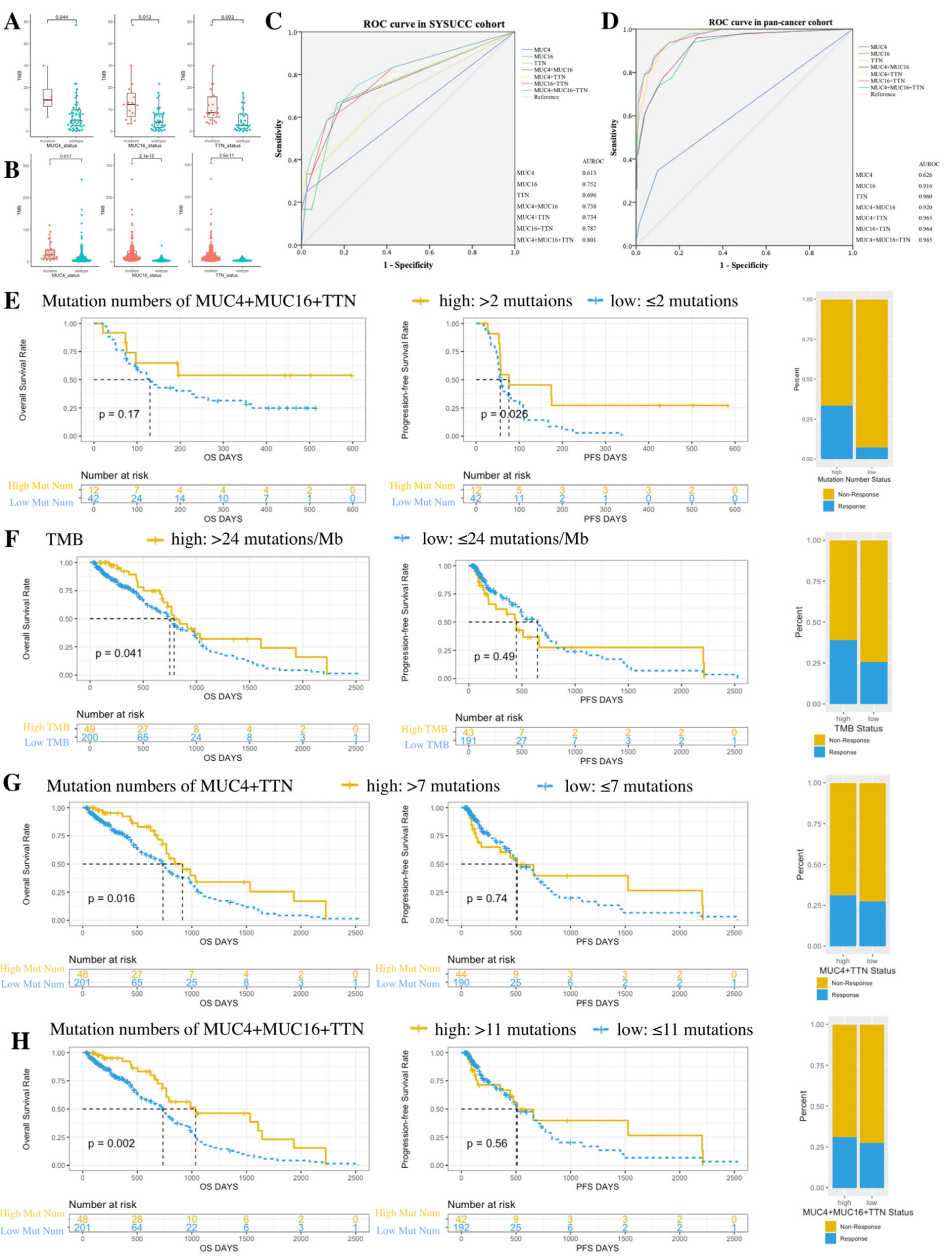
Mutation status and mutation number of *MUC4*, *MUC16*, and *TTN* predict TMB, prognosis, and immunotherapy efficacy in SYSUCC and pan‐cancer cohort. A and B, *MUC4*, *MUC16*, and *TTN* mutations had higher TMB in SYSUCC and pan‐cancer cohort. C and D, ROC curve using gene mutation numbers to estimate TMB status. E, High mutation numbers of *MUC4*, *MUC16*, and *TTN* genes showed better OS, PFS, and immunotherapy efficacy in SYSUCC cohort. F‐H, TMB status, mutation numbers of *MUC4* plus *TTN*, and mutation numbers of *MUC4*, *MUC16* plus *TTN* were correlated with OS, PFS, and ORR in pan‐cancer cohort

In conclusion, we found *MUC4* and *MUC16* mutations were potentially associated with prognosis, while H4205Q and V3305_S3320del mutation of MUC4 might be functional mutation sites that affect tumor progression and prognosis. Mutation status and mutation numbers of *MUC4*, *MUC16*, and *TTN* showed high potency in predicting TMB. Random mutation of long gene had no specific function, but might become window to represent whole TMB. Single long gene mutation showed certain but limited correlation with TMB, but combination of *MUC4*, *MUC16*, and *TTN* showed better correlation with TMB and was sufficient to predict TMB. We put forward a gene combination, *MUC4*, *MUC16*, and *TTN*, to predict TMB, and this gene combination may serve as a more economic and convenient biomarker for immunotherapy efficacy substituting for TMB.

## ETHICS APPROVAL

For FUSCC cohort, approval was granted by the Ethics Committee of Fudan University Shanghai Cancer Center, and signed informed consents were obtained from all participants. For SYSUCC cohort, the study protocol was approved by the institutional ethics committees of all participating centers, and signed informed consents were obtained from all participants.

## CONFLICT OF INTEREST

The authors declared no conflict of interest.

## AUTHOR CONTRIBUTIONS

Conceptualization: Guo; project administration and resource: Guo, Xu, and Zhao; data curation: Yang, Zhang, Chen, Xu, and Zhao; formal analysis: Yang, Zhang, Chen, and Zhao; writing and original draft: Yang; writing and review and editing: Guo.

## Supporting information

Supporting Information

Supporting Information

Supporting Information

Supporting Information

Supporting Information

Supporting Information

Supporting Information

Supporting Information

Supporting Information

Supporting Information

## Data Availability

The FUSCC and SYSUCC datasets generated and/or analyzed during the current study are not publicly available due information protection but are available from the corresponding author on reasonable request. The pan‐cancer dataset generated and/or analyzed during the current study are available in the reference.^8^
