# Supplementary figures and images for: MUC4, MUC16, and TTN genes mutation correlated with prognosis, and predicted tumor mutation burden and immunotherapy efficacy in gastric cancer and pan‐cancer
Source: Clin Transl Med. 2020 Aug 22;10(4):e155. doi: 10.1002/ctm2.155 (PMC7443139; doi:10.1002/ctm2.155)

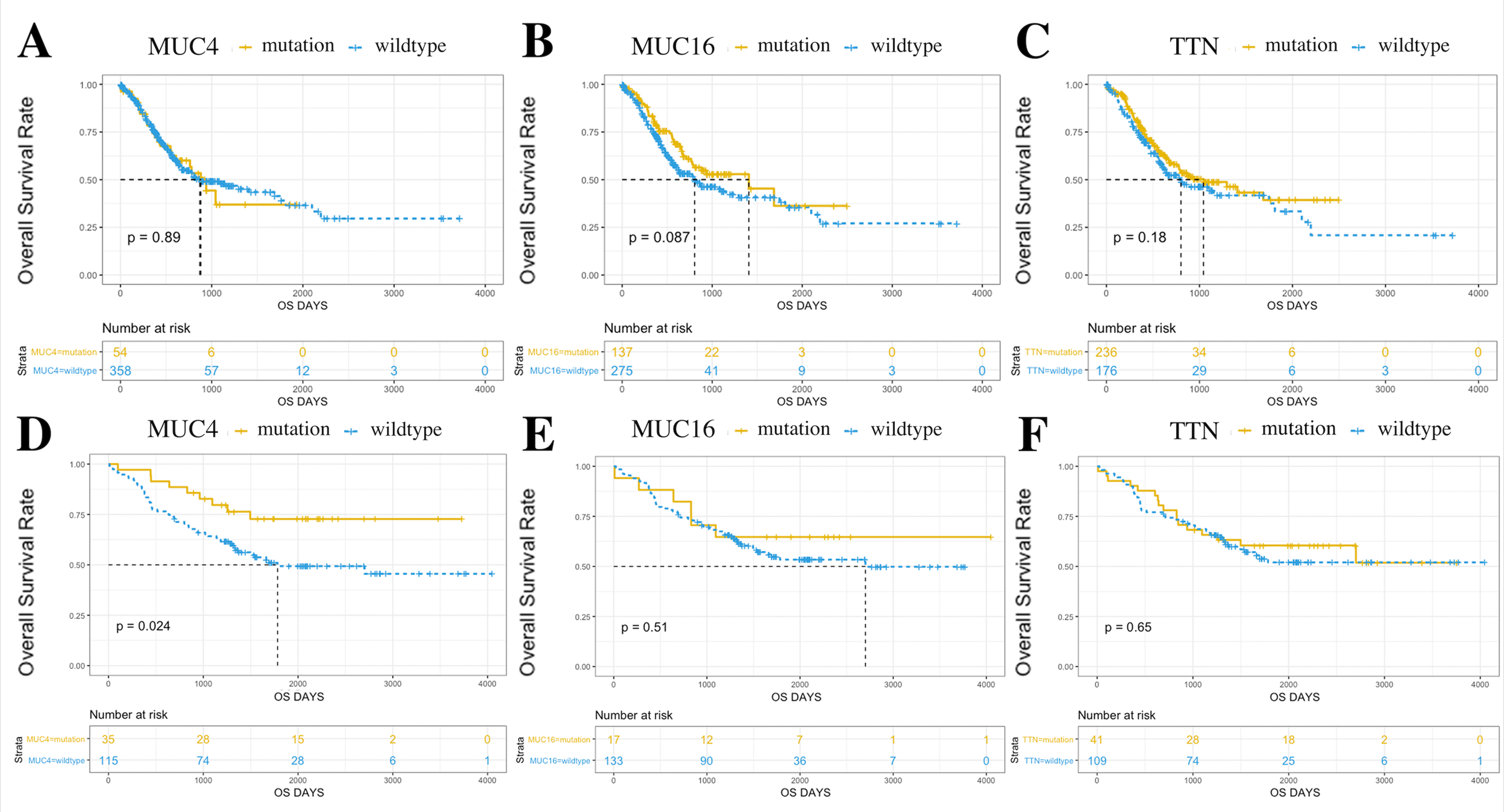

Supplement: Supplementary file 1 — Supporting Information [file CTM2-10-e155-s001.tiff]

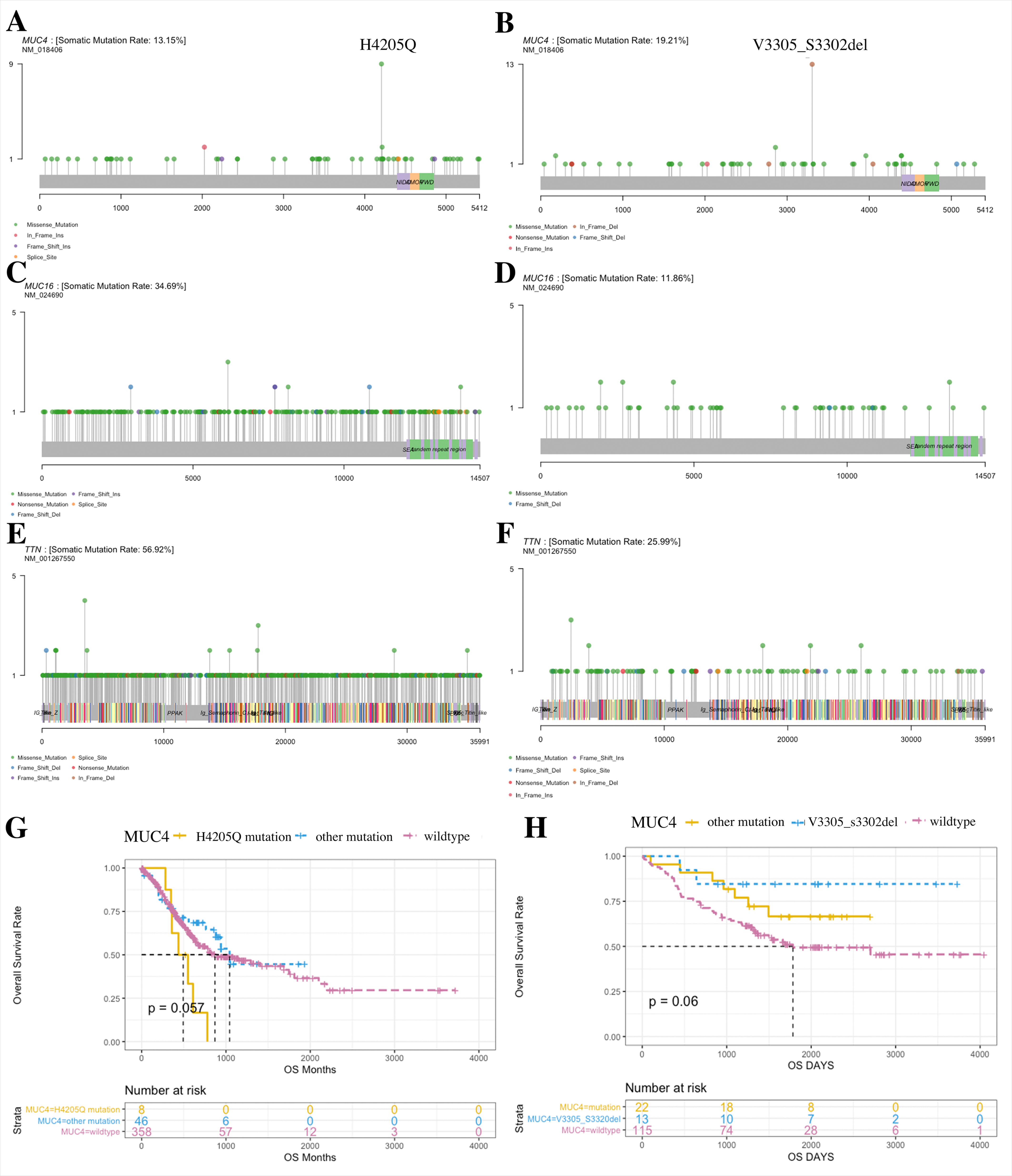

Supplement: Supplementary file 2 — Supporting Information [file CTM2-10-e155-s002.tiff]

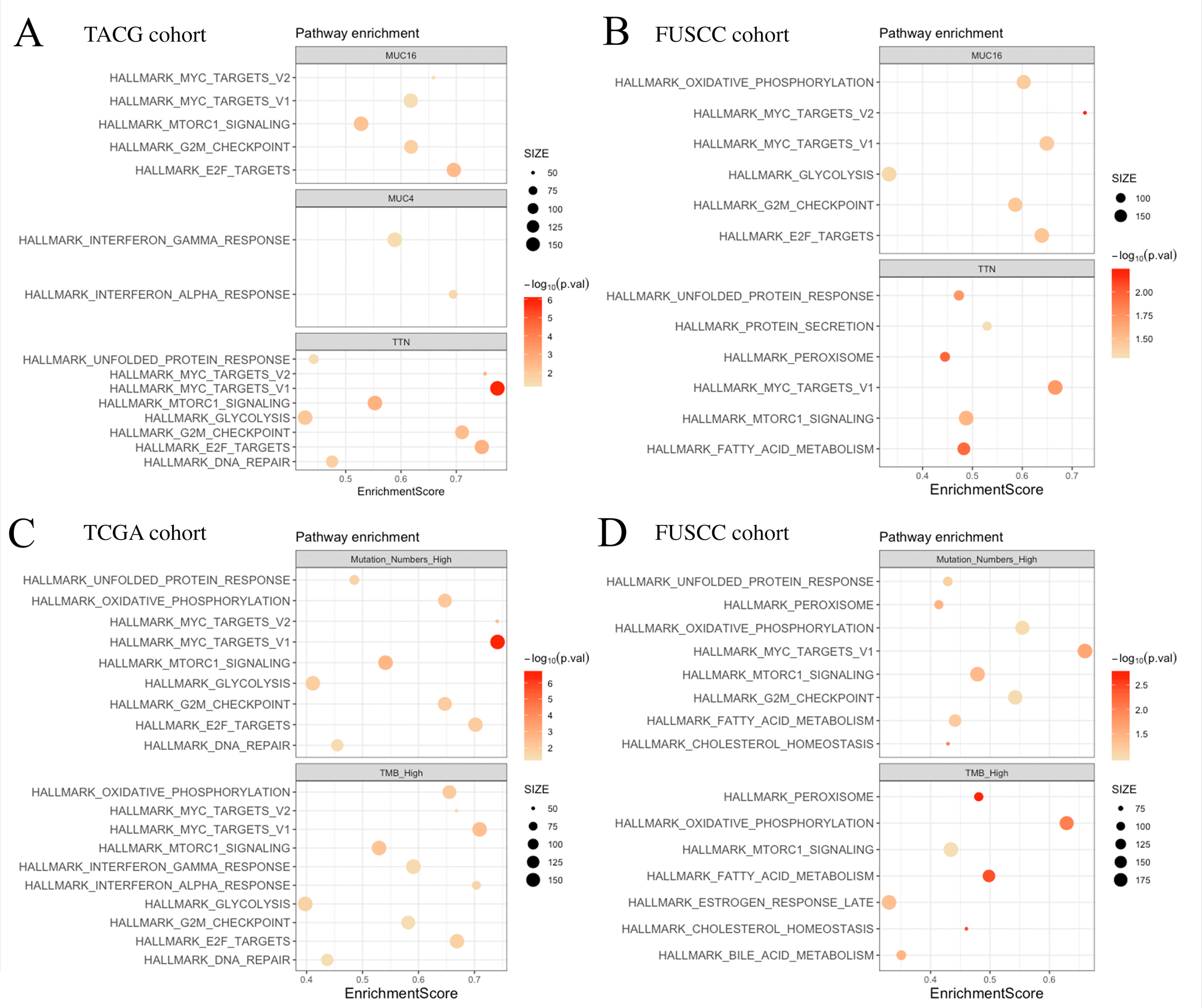

Supplement: Supplementary file 3 — Supporting Information [file CTM2-10-e155-s003.tiff]

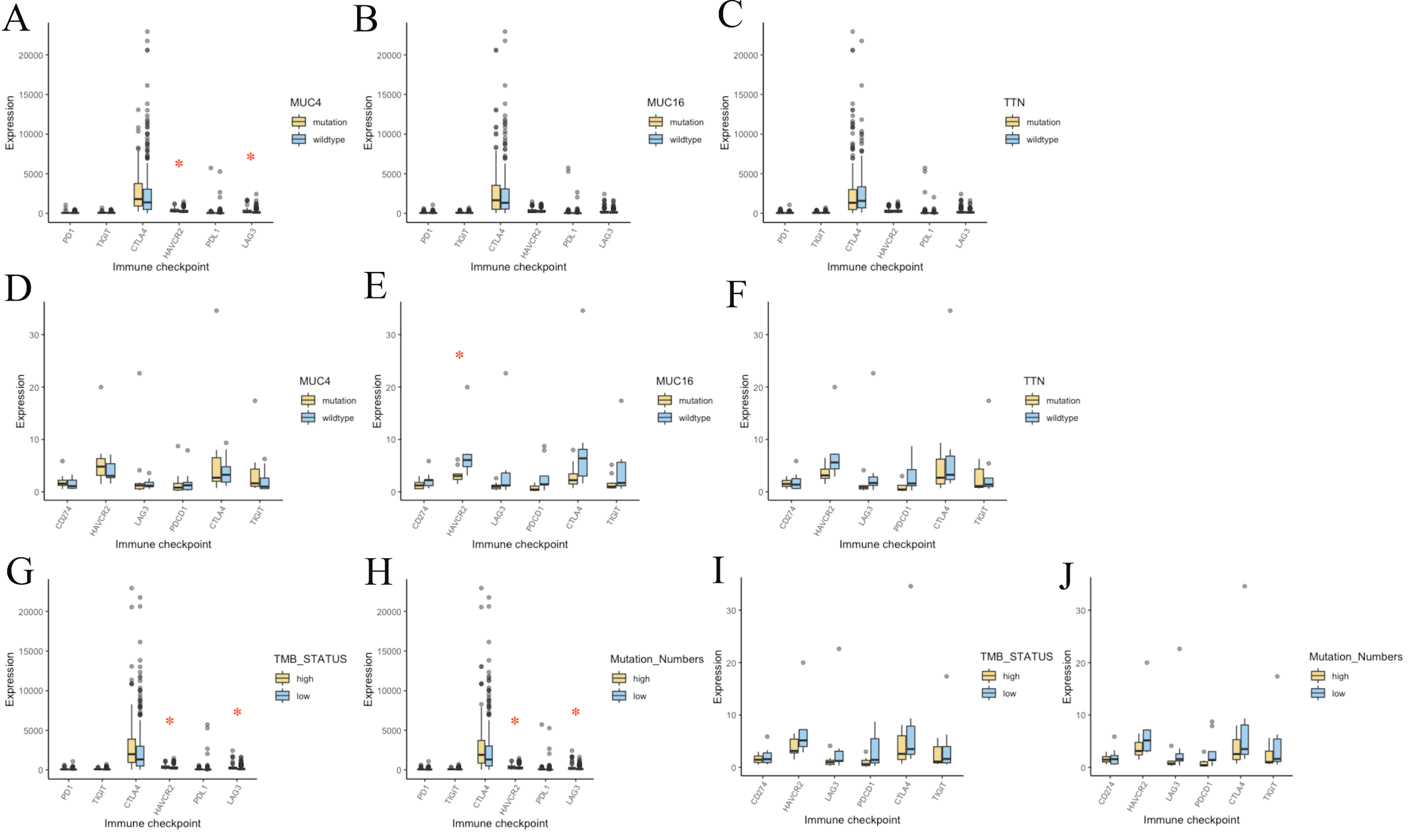

Supplement: Supplementary file 4 — Supporting Information [file CTM2-10-e155-s004.tiff]

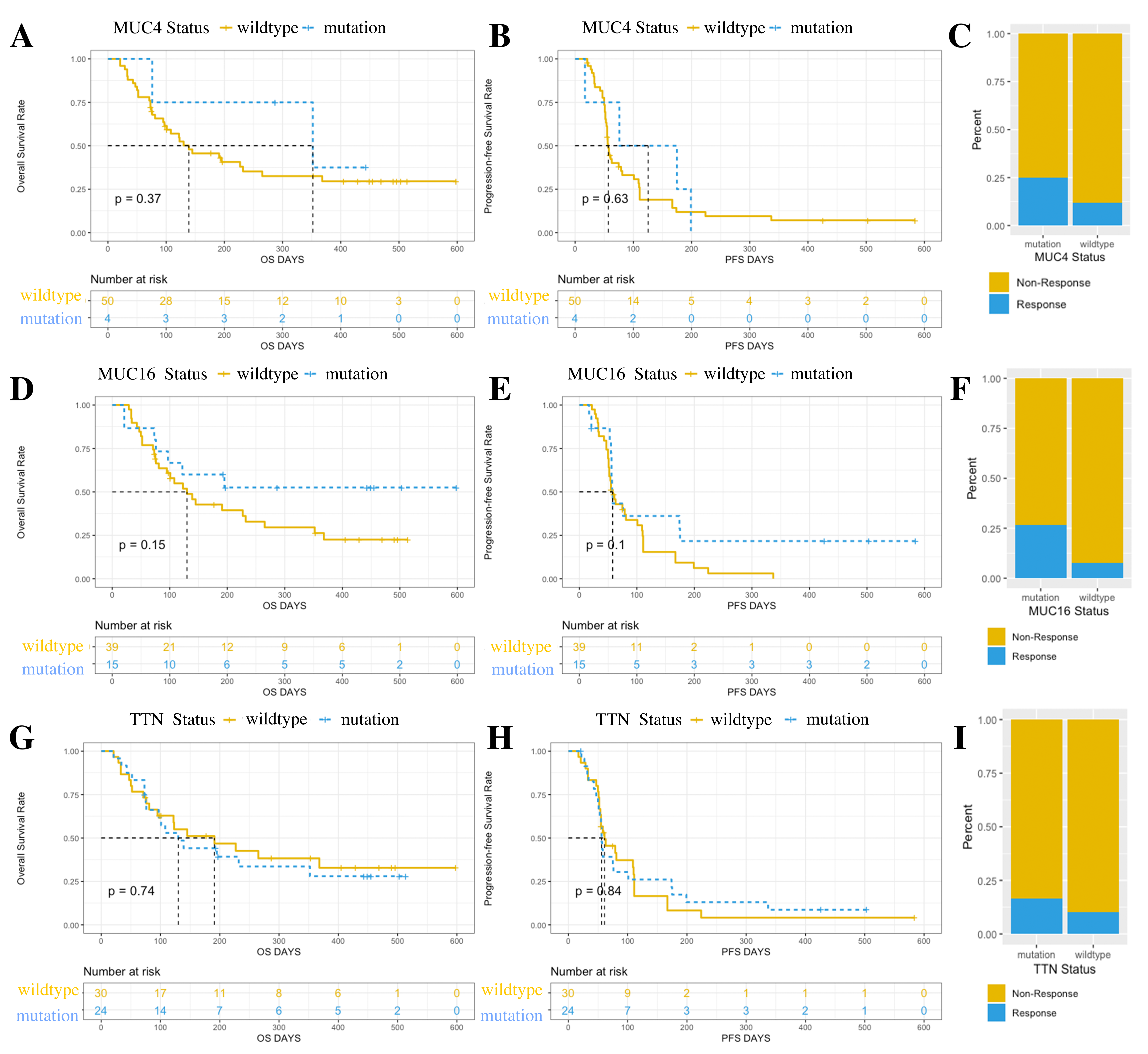

Supplement: Supplementary file 8 — Supporting Information [file CTM2-10-e155-s008.tif]

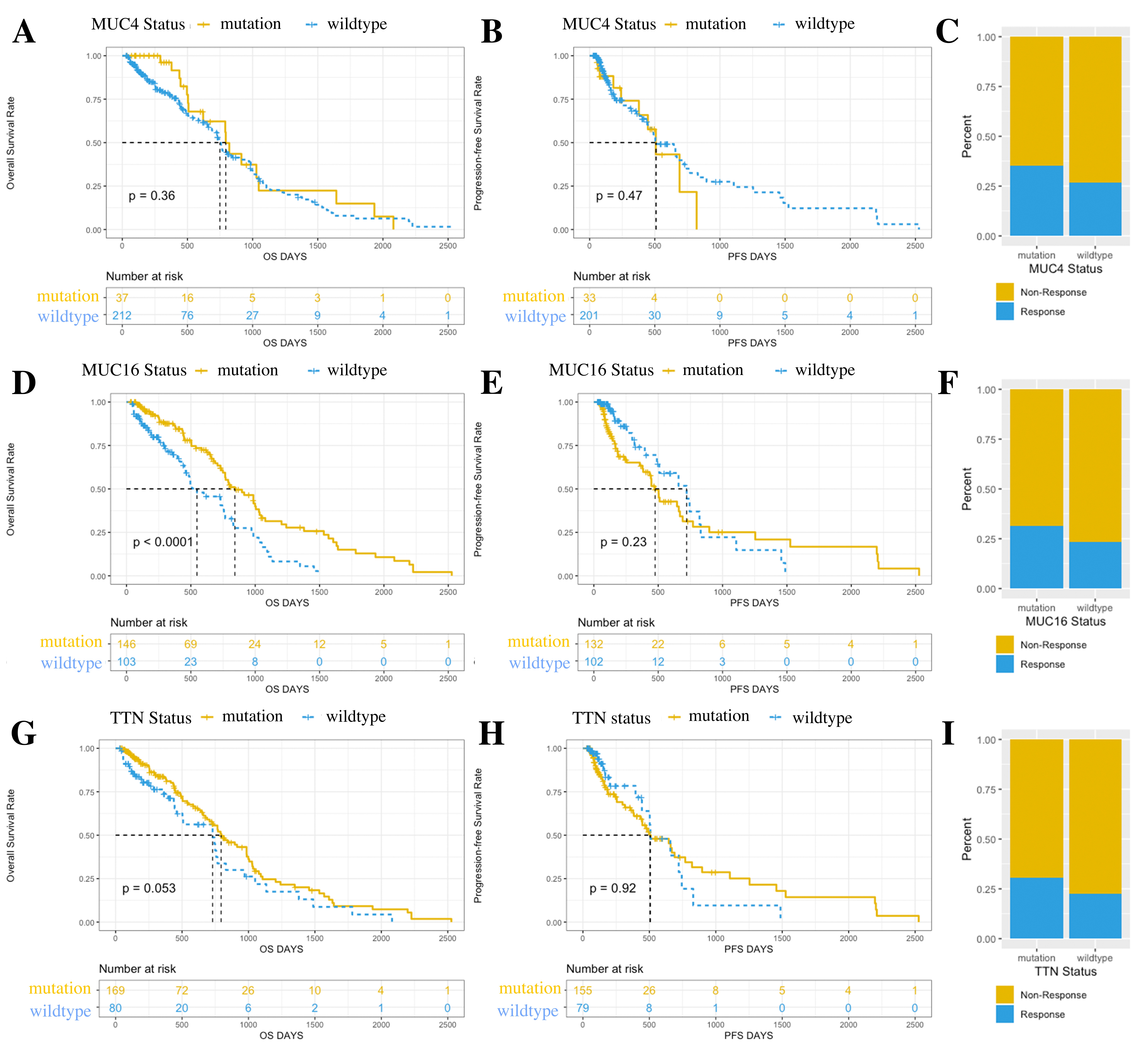

Supplement: Supplementary file 9 — Supporting Information [file CTM2-10-e155-s009.tif]
