# Supplementary material for: MUC4, MUC16, and TTN genes mutation correlated with prognosis, and predicted tumor mutation burden and immunotherapy efficacy in gastric cancer and pan‐cancer
Source: Clin Transl Med. 2020 Aug 22;10(4):e155. doi: 10.1002/ctm2.155 (PMC7443139; doi:10.1002/ctm2.155)
Supplement: Supplementary file 10 — Supporting Information [file CTM2-10-e155-s010.docx]

**Supplementary Table**

**Table S1: Length, mutation rate and correlation with prognosis of Mucin genes in TCGA and FUSCC cohort.**

|  |  | TCGA cohort | | FUSCC cohort | |
| --- | --- | --- | --- | --- | --- |
| Gene | **Length** | **Mutation rate** | **OS p value** | **Mutation rate** | **OS p value** |
| MUC1 | 7092bp | 1.81% (8/441) | P=0.016 | 1.69% (3/177) | P=0.64 |
| MUC2 | 37531bp | 7.48% (33/441) | P=0.37 | 5.65% (10/177) | P=0.18 |
| MUC3A | 18814bp | 3.63% (16/441) | P=0.63 | 1.13% (2/177) | P=0.61 |
| MUC4 | 65159bp | 13.15% (58/441) | P=0.89 | 19.77% (35/177) | P=0.024 |
| MUC5AC | 43186bp | 1.81% (8/441) | P=0.33 | 0.00% (0/177) | / |
| MUC5B | 39107bp | 11.79% (52/441) | P=0.21 | 8.47% (15/177) | P=0.83 |
| MUC6 | 23896bp | 15.87% (70/441) | P=0.59 | 5.08% (9/177) | P=0.86 |
| MUC7 | 52506bp | 2.49% (11/441) | P=0.75 | 1.13% (2/177) | P=0.088 |
| MUC8 | 5068bp | / |  | 3.39% (6/177) | P=0.36 |
| MUC12 | 49327bp | 0.23% (1/441) | P=0.018 | 8.47% (15/177) | P=0.19 |
| MUC13 | 29310bp | 0.68% (3/441) | P=0.63 | 1.69% (3/177) | P=0.4 |
| MUC15 | 13232bp | 3.63% (16/441) | P=0.3 | 2.26% (4/177) | P=0.77 |
| MUC16 | 132499bp | 34.69% (153/441) | P=0.087 | 11.86% (21/177) | P=0.51 |
| MUC17 | 38779bp | 16.33% (72/441) | P=0.46 | 3.95% (7/177) | P=0.89 |
| MUC19 | 177364bp | / |  | 3.39% (6/177) | P=0.35 |
| MUC20 | 12574bp | 1.81% (8/441) | P=0.69 | 2.26% (4/177) | P=0.67 |
| MUC21 | 6186bp | 1.36% (6/441) | P=1 | 3.39% (6/177) | P=0.71 |

**Table S2: Correlation coefficient of gene mutation number and TMB in TCGA, FUSCC, SYSUCC and pan-cancer cohort.**

|  | TCGA | | FUSCC | | SYSUCC | | | Pan-cancer | | |
| --- | --- | --- | --- | --- | --- | --- | --- | --- | --- | --- |
|  | ρ | P value | ρ | P value | | ρ | P value | ρ | P value |  |
| MUC4 | 0.373 | <0.001 | 0.403 | <0.001 | | 0.279 | 0.041 | 0.336 | <0.001 |  |
| MUC16 | 0.560 | <0.001 | 0.589 | <0.001 | | 0.475 | <0.001 | 0.749 | <0.001 |  |
| TTN | 0.711 | <0.001 | 0.704 | <0.001 | | 0.517 | <0.001 | 0.790 | <0.001 |  |
| MUC4+MUC16 | 0.615 | <0.001 | 0.604 | <0.001 | | 0.478 | <0.001 | 0.762 | <0.001 |  |
| MUC4+TTN | 0.743 | <0.001 | 0.681 | <0.001 | | 0.548 | <0.001 | 0.804 | <0.001 |  |
| MUC16+TTN | 0.764 | <0.001 | 0.748 | <0.001 | | 0.599 | <0.001 | 0.819 | <0.001 |  |
| MUC4+MUC16+TTN | 0.782 | <0.001 | 0.735 | <0.001 | | 0.607 | <0.001 | 0.827 | <0.001 |  |

**Table S3: Youden index of different mutation numbers in TCGA cohort.**

| Mutation numbers of MUC4+MUC16+TTN | Youden index |
| --- | --- |
| 0.5 | 0.338 |
| 1.5 | 0.551 |
| 2.5 | 0.688 |
| 3.5 | 0.723 |
| 4.5(cutoff value) | 0.769 |
| 5.5 | 0.661 |
| 6.5 | 0.556 |
| 7.5 | 0.498 |
| 8.5 | 0.436 |
| 9.5 | 0.381 |

**Table S4: Youden index of different mutation numbers in FUSCC cohort.**

| Mutation numbers | Youden index for MUC16+TTN | Youden index for MUC4+MUC16+TTN |
| --- | --- | --- |
| 0.5 | 0.717 | 0.575 |
| 1.5 | 0.699 (cutoff value) | 0.712 |
| 2.5 | 0.561 | 0.61 (cutoff value) |
| 3.5 | 0.479 | 0.548 |
| 4.5 | 0.417 | 0.5 |
| 5.5 | 0.333 | 0.417 |

**Table S5: univariate and multivariate analysis of overall survival in TCGA cohort.**

| **Variable** | **Univariate analysis** | **Multivariate analysis** | | | | |  |
| --- | --- | --- | --- | --- | --- | --- | --- |
|  | **P value** | **HR (95%CI)** | | **P value** | | |  |
| Age | 0.028 |  | | | |  |  |
| <65 |  | Reference | | | |  |  |
| ≥65 |  | 1.884 (1.343-2.641) | | | | <0.001 |  |
| Gender | 0.55 |  | | | |  |  |
| Female |  |  | | | |  |  |
| male |  |  | | | |  |  |
| T stage ^*^ | 0.0054 |  | | | | 0.042 |  |
| T1 |  | Reference | | | |  |  |
| T2 |  | 4.754 (0.635-35.611) | | | | 0.129 |  |
| T3 |  | 4.587 (0.624-33.702) | | | | 0.134 |  |
| T4 |  | 6.961 (0.947-51.175) | | | | 0.057 |  |
| N stage ^*^ | <0.001 |  | | | | 0.002 |  |
| N0 |  | Reference | | | |  |  |
| N1 |  | 1.535 (0.939-2.510) | | | | 0.087 |  |
| N2 |  | 1.388 (0.827-2.332) | | | | 0.215 |  |
| N3 |  | 2.491 (1.535-4.043) | | | | <0.001 |  |
| M stage ^*^ | <0.001 |  | | | |  |  |
| M0 |  | Reference | | | |  |  |
| M1 |  | 2.483 (1.395-4.421) | | | | 0.002 |  |
| Histology | 0.0076 |  | | | | 0.052 |  |
| Other adenocarcinoma |  | Reference | | | |  |  |
| Mucinous adenocarcinoma |  | 0.239 (0.076-0.757) | | | | 0.015 |  |
| Signet ring cell carcinoma |  | 0.978 (0.433-2.209) | | | | 0.957 |  |
| Histological grade | 0.24 |  | | | |  |  |
| G1 |  |  | | | |  |  |
| G2 |  |  | | | |  |  |
| G3 |  |  | | | |  |  |
| MUC4 mutation types | 0.057 |  | | | | 0.091 |  |
| Wildtype |  | Reference | | | |  |  |
| Other mutation |  | 2.266 (1.028-4.994) | | | | 0.043 |  |
| H4205Q |  | 0.797 (0.416-1.529) | | | | 0.495 |  |
| MUC16 mutation status | 0.087 | |  | |  | | |
| Wildtype |  | | Reference | |  | | |
| Mutation |  | | 0.824 (0.541-1.253) | | 0.364 | | |
| TTN mutation status | 0.18 | |  | |  | | |
| Wildtype |  | |  | |  | | |
| Mutation |  | |  | |  | | |
| TMB | 0.045 | |  | |  | | |
| High (>20 Muts/Mb) |  | | Reference | |  | | |
| Low (≤20 Muts/Mb) |  | | 1.625 (0.829-3.183) | | 0.157 | | |
| Mutation numbers of MUC4, MUC16 and TTN | 0.027 | |  | |  | | |
| High (>4 mutations) |  | | Reference | |  | | |
| Low (≤4 mutations) |  | | 1.078 (0.543-2.14) | | 0.830 | | |

* T, N, M stages were adjusted by AJCC 7^th^ edition.

**Table S6: univariate and multivariate analysis of overall survival in FUSCC cohort.**

| **Variable** | **Univariate analysis** | **Multivariate analysis** | | |
| --- | --- | --- | --- | --- |
|  | **P value** | **HR (95%CI)** | **P value** | |
| Age | 0.034 |  | |  |
| <65 |  | Reference | |  |
| ≥65 |  | 1.513 (0.908-2.523) | | 0.112 |
| Gender | 0.18 |  | |  |
| Female |  |  | |  |
| male |  |  | |  |
| T stage ^*^ | <0.001 |  | | 0.003 |
| T1 |  | Reference | |  |
| T2 |  | 1.756 (0.405-7.606) | | 0.452 |
| T3 |  | 5.151 (1.998-13.281) | | <0.001 |
| T4 |  | 4.861 (1.578-14.974) | | 0.006 |
| N stage ^*^ | <0.001 |  | | 0.048 |
| N0 |  | Reference | |  |
| N1 |  | 1.277 (0.466-3.498) | | 0.635 |
| N2 |  | 2.31 (0.946-5.639) | | 0.066 |
| N3 |  | 2.741 (1.236-6.081) | | 0.013 |
| M stage ^*^ | 0.93 |  | |  |
| M0 |  |  | |  |
| M1 |  |  | |  |
| Histology | 0.8 |  | |  |
| Other adenocarcinoma |  |  | |  |
| Mucinous adenocarcinoma |  |  | |  |
| Signet ring cell carcinoma |  |  | |  |
| Histological grade | 0.2 |  | |  |
| G1 |  |  | |  |
| G2 |  |  | |  |
| G3 |  |  | |  |
| G4 |  |  | |  |
| MUC4 mutation types | 0.06 |  | | 0.057 |
| Wildtype |  | Reference | |  |
| Other mutation |  | 1.682 (0.640-4.419) | | 0.291 |
| V3305_S3320del |  | 0.221 (0.053-0.928) | | 0.039 |
| MUC16 mutation status | 0.51 |  | |  |
| Wildtype |  |  | |  |
| Mutation |  |  | |  |
| TTN mutation status | 0.65 |  | |  |
| Wildtype |  |  | |  |
| Mutation |  |  | |  |
| TMB | 0.0023 |  | |  |
| High (>8 Muts/Mb) |  | Reference | |  |
| Low (≤8 Muts/Mb) |  | 2.123 (0.763-5.908) | | 0.149 |
| Mutation numbers of MUC4, MUC16 and TTN | 0.22 |  | |  |
| High (>2 mutations) |  |  | |  |
| Low (≤2 mutations) |  |  | |  |

* T, N, M stages were adjusted by AJCC 7^th^ edition.

**Table S7: Gene mutation status of MUC4, MUC16 and TTN, TMB and mutation numbers showed association with clinicopathological characteristics.**

| **TCGA cohort** | | MUC4 | | | MUC4_status | | | MUC16 | | TTN | | TMB | | | MUCTTN | |
| --- | --- | --- | --- | --- | --- | --- | --- | --- | --- | --- | --- | --- | --- | --- | --- | --- |
|  |  | mutation | | wildtype | H4205Q | other mutation | wildtype | mutation | wildtype | mutation | wildtype | high | low | | high | low |
| AJCC_T | T1 | 2 | | 21 | 0 | 2 | 21 | 13 | 10 | 16 | 7 | 8 | 15 | | 8 | 15 |
|  |  | 3.40% | | 5.50% | 0.00% | 4.10% | 5.50% | 8.50% | 3.50% | 6.40% | 3.70% | 9.30% | 4.20% | | 8.30% | 4.30% |
|  | T2 | 17 | | 61 | 2 | 15 | 61 | 25 | 53 | 46 | 32 | 14 | 64 | | 20 | 58 |
|  |  | 29.30% | | 15.90% | 22.20% | 30.60% | 15.90% | 16.30% | 18.40% | 18.30% | 16.80% | 16.30% | 18.00% | | 20.80% | 16.80% |
|  | T3 | 13 | | 141 | 2 | 11 | 141 | 52 | 102 | 83 | 71 | 22 | 132 | | 20 | 134 |
|  |  | 22.40% | | 36.80% | 22.20% | 22.40% | 36.80% | 34.00% | 35.40% | 33.10% | 37.40% | 25.60% | 37.20% | | 20.80% | 38.80% |
|  | T4 | 22 | | 155 | 5 | 17 | 155 | 58 | 119 | 101 | 76 | 38 | 139 | | 44 | 133 |
|  |  | 37.90% | | 40.50% | 55.60% | 34.70% | 40.50% | 37.90% | 41.30% | 40.20% | 40.00% | 44.20% | 39.20% | | 45.80% | 38.60% |
|  | Tx | 4 | | 5 | 0 | 4 | 5 | 5 | 4 | 5 | 4 | 4 | 5 | | 4 | 5 |
|  |  | 6.90% | | 1.30% | 0.00% | 8.20% | 1.30% | 3.30% | 1.40% | 2.00% | 2.10% | 4.70% | 1.40% | | 4.20% | 1.40% |
|  |  |  | | p=0.003 |  |  | p=0.035 |  | p=0.128 |  | p=0.696 |  | p=0.037 | |  | p=0.008 |
|  |  | mutation | | wildtype | H4205Q | other mutation | wildtype | mutation | wildtype | mutation | wildtype | high | low | | high | low |
| AJCC_N | N0 | 17 | | 115 | 3 | 14 | 115 | 51 | 81 | 79 | 53 | 36 | 96 | | 36 | 96 |
|  |  | 29.30% | | 30.00% | 33.30% | 28.60% | 30.00% | 33.30% | 28.10% | 31.50% | 27.90% | 41.90% | 27.00% | | 37.50% | 27.80% |
|  | N1 | 15 | | 102 | 2 | 13 | 102 | 46 | 71 | 61 | 56 | 24 | 93 | | 29 | 88 |
|  |  | 25.90% | | 26.60% | 22.20% | 26.50% | 26.60% | 30.10% | 24.70% | 24.30% | 29.50% | 27.90% | 26.20% | | 30.20% | 25.50% |
|  | N2 | 11 | | 74 | 3 | 8 | 74 | 28 | 57 | 52 | 33 | 11 | 74 | | 16 | 69 |
|  |  | 19.00% | | 19.30% | 33.30% | 16.30% | 19.30% | 18.30% | 19.80% | 20.70% | 17.40% | 12.80% | 20.80% | | 16.70% | 20.00% |
|  | N3 | 10 | | 78 | 1 | 9 | 78 | 17 | 71 | 50 | 38 | 10 | 78 | | 9 | 79 |
|  |  | 17.20% | | 20.40% | 11.10% | 18.40% | 20.40% | 11.10% | 24.70% | 19.90% | 20.00% | 11.60% | 22.00% | | 9.40% | 22.90% |
|  | Nx | 5 | | 14 | 0 | 5 | 14 | 11 | 8 | 9 | 10 | 5 | 14 | | 6 | 13 |
|  |  | 8.60% | | 3.70% | 0.00% | 10.20% | 3.70% | 7.20% | 2.80% | 3.60% | 5.30% | 5.80% | 3.90% | | 6.30% | 3.80% |
|  |  |  | | p=0.533 |  |  | p=0.598 |  | p=0.003 |  | p=0.577 |  | p=0.019 | |  | p=0.023 |
|  |  | mutation | | wildtype | H4205Q | other mutation | wildtype | mutation | wildtype | mutation | wildtype | high | low | | high | low |
| AJCC_M | M0 | 53 | | 336 | 8 | 45 | 336 | 137 | 252 | 223 | 166 | 77 | 312 | | 89 | 300 |
|  |  | 91.40% | | 87.70% | 88.90% | 91.80% | 87.70% | 89.50% | 87.50% | 88.80% | 87.40% | 89.50% | 87.90% | | 92.70% | 87.00% |
|  | M1 | 2 | | 28 | 0 | 2 | 28 | 9 | 21 | 13 | 17 | 5 | 25 | | 3 | 27 |
|  |  | 3.40% | | 7.30% | 0.00% | 4.10% | 7.30% | 5.90% | 7.30% | 5.20% | 8.90% | 5.80% | 7.00% | | 3.10% | 7.80% |
|  | Mx | 3 | | 19 | 1 | 2 | 19 | 7 | 15 | 15 | 7 | 4 | 18 | | 4 | 18 |
|  |  | 5.20% | | 5.00% | 11.10% | 4.10% | 5.00% | 4.60% | 5.20% | 6.00% | 3.70% | 4.70% | 5.10% | | 4.20% | 5.20% |
|  |  |  | | p=0.498 |  |  | p=0.616 |  | p=0.811 |  | p=0.181 |  | p=0.905 | |  | p=0.236 |
|  |  | mutation | | wildtype | H4205Q | other mutation | wildtype | mutation | wildtype | mutation | wildtype | high | low | | high | low |
| AJCC_Stage | Stage I | 7 | | 49 | 1 | 6 | 49 | 20 | 36 | 34 | 22 | 15 | 41 | | 17 | 39 |
|  |  | 13.50% | | 13.10% | 11.10% | 14.00% | 13.10% | 13.90% | 12.80% | 14.10% | 11.90% | 19.00% | 11.80% | | 19.10% | 11.60% |
|  | Stage II | 19 | | 129 | 3 | 16 | 129 | 56 | 92 | 83 | 65 | 31 | 117 | | 33 | 115 |
|  |  | 36.50% | | 34.50% | 33.30% | 37.20% | 34.50% | 38.90% | 32.60% | 34.40% | 35.10% | 39.20% | 33.70% | | 37.10% | 34.10% |
|  | Stage III | 24 | | 168 | 5 | 19 | 168 | 59 | 133 | 111 | 81 | 28 | 164 | | 36 | 156 |
|  |  | 46.20% | | 44.90% | 55.60% | 44.20% | 44.90% | 41.00% | 47.20% | 46.10% | 43.80% | 35.40% | 47.30% | | 40.40% | 46.30% |
|  | Stage IV | 2 | | 28 | 0 | 2 | 28 | 9 | 21 | 13 | 17 | 5 | 25 | | 3 | 27 |
|  |  | 3.80% | | 7.50% | 0.00% | 4.70% | 7.50% | 6.30% | 7.40% | 5.40% | 9.20% | 6.30% | 7.20% | | 3.40% | 8.00% |
|  |  |  | | p=0.817 |  |  | p=0.909 |  | p=0.537 |  | p=0.446 |  | p=0.161 | |  | p=0.117 |
|  |  | mutation | | wildtype | H4205Q | other mutation | wildtype | mutation | wildtype | mutation | wildtype | high | low | | high | low |
| Histology | Adenocarcinoma | 56 | | 349 | 8 | 48 | 349 | 140 | 265 | 229 | 176 | 79 | 326 | | 90 | 315 |
|  |  | 96.60% | | 91.10% | 88.90% | 98.00% | 91.10% | 91.50% | 92.00% | 91.20% | 92.60% | 91.90% | 91.80% | | 93.80% | 91.30% |
|  | Mucious | 1 | | 21 | 0 | 1 | 21 | 8 | 14 | 14 | 8 | 4 | 18 | | 5 | 17 |
|  |  | 1.70% | | 5.50% | 0.00% | 2.00% | 5.50% | 5.20% | 4.90% | 5.60% | 4.20% | 4.70% | 5.10% | | 5.20% | 4.90% |
|  | Signet ring cell | 1 | | 13 | 1 | 0 | 13 | 5 | 9 | 8 | 6 | 3 | 11 | | 1 | 13 |
|  |  | 1.70% | | 3.40% | 11.10% | 0.00% | 3.40% | 3.30% | 3.10% | 3.20% | 3.20% | 3.50% | 3.10% | | 1.00% | 3.80% |
|  |  |  | | p=0.283 |  |  | p=0.155 |  | p=0.982 |  | p=0.807 |  | p=0.972 | |  | p=0.319 |
|  |  | mutation | | wildtype | H4205Q | other mutation | wildtype | mutation | wildtype | mutation | wildtype | high | low | | high | low |
| GRADE | G1 | 3 | | 9 | 1 | 2 | 9 | 8 | 4 | 9 | 3 | 3 | 9 | | 4 | 8 |
|  |  | 5.20% | | 2.40% | 11.10% | 4.10% | 2.40% | 5.40% | 1.40% | 3.70% | 1.60% | 3.50% | 2.60% | | 4.20% | 2.40% |
|  | G2 | 22 | | 137 | 4 | 18 | 137 | 55 | 104 | 87 | 72 | 28 | 131 | | 34 | 125 |
|  |  | 37.90% | | 36.60% | 44.40% | 36.70% | 36.60% | 36.90% | 36.70% | 35.40% | 38.70% | 32.90% | 37.80% | | 35.80% | 37.10% |
|  | G3 | 33 | | 228 | 4 | 29 | 228 | 86 | 175 | 150 | 111 | 54 | 207 | | 57 | 204 |
|  |  | 56.90% | | 61.00% | 44.40% | 59.20% | 61.00% | 57.70% | 61.80% | 61.00% | 59.70% | 63.50% | 59.70% | | 60.00% | 60.50% |
|  |  |  | | p=0.462 |  |  | p=0.678 |  | p=0.056 |  | p=0.378 |  | p=0.666 | |  | p=0.626 |
| **FUSCC cohort** | | MUC4 |  | | MUC4_STATUS | | | MUC16 | | TTN | | TMB | |  | MUCTTN | |
|  |  | mutation | | wildtype | wildtype | other mutation | V3305_S3320del | mutation | wildtype | mutation | wildtype | high | low | | high | low |
| AJCC_T | T1 | 19 | | 24 | 24 | 16 | 3 | 10 | 33 | 23 | 20 | 23 | 20 | | 17 | 26 |
|  |  | 54.30% | | 17.00% | 17.00% | 72.70% | 23.10% | 47.60% | 21.30% | 50.00% | 15.40% | 63.90% | 14.30% | | 53.10% | 18.10% |
|  | T2 | 2 | | 14 | 14 | 0 | 2 | 3 | 13 | 3 | 13 | 2 | 14 | | 4 | 12 |
|  |  | 5.70% | | 9.90% | 9.90% | 0.00% | 15.40% | 14.30% | 8.40% | 6.50% | 10.00% | 5.60% | 10.00% | | 12.50% | 8.30% |
|  | T3 | 14 | | 80 | 80 | 6 | 8 | 6 | 88 | 15 | 79 | 8 | 86 | | 9 | 85 |
|  |  | 40.00% | | 56.70% | 56.70% | 27.30% | 61.50% | 28.60% | 56.80% | 32.60% | 60.80% | 22.20% | 61.40% | | 28.10% | 59.00% |
|  | T4 | 0 | | 23 | 23 | 0 | 0 | 2 | 21 | 5 | 18 | 3 | 20 | | 2 | 21 |
|  |  | 0.00% | | 16.30% | 16.30% | 0.00% | 0.00% | 9.50% | 13.50% | 10.90% | 13.80% | 8.30% | 14.30% | | 6.30% | 14.60% |
|  |  |  | | p<0.001 |  |  | p=0.001 |  | p=0.039 |  | p<0.001 |  | p<0.001 | |  | p<0.001 |
|  |  | mutation | | wildtype | wildtype | other mutation | V3305_S3320del | mutation | wildtype | mutation | wildtype | high | low | | high | low |
| AJCC_N | N0 | 10 | | 26 | 26 | 7 | 3 | 8 | 28 | 8 | 28 | 9 | 27 | | 9 | 27 |
|  |  | 28.60% | | 18.44% | 18.40% | 31.80% | 23.10% | 38.10% | 18.10% | 17.40% | 21.50% | 25.00% | 19.30% | | 28.10% | 18.80% |
|  | N1 | 8 | | 34 | 34 | 5 | 3 | 6 | 36 | 16 | 26 | 13 | 29 | | 10 | 32 |
|  |  | 22.90% | | 24.11% | 24.10% | 22.70% | 23.10% | 28.60% | 23.20% | 34.80% | 20.00% | 36.10% | 20.70% | | 31.30% | 22.20% |
|  | N2 | 1 | | 30 | 30 | 0 | 1 | 0 | 31 | 5 | 26 | 5 | 26 | | 1 | 30 |
|  |  | 2.90% | | 21.28% | 21.30% | 0.00% | 7.70% | 0.00% | 20.00% | 10.90% | 20.00% | 13.90% | 18.60% | | 3.10% | 20.80% |
|  | N3 | 16 | | 51 | 51 | 10 | 6 | 7 | 60 | 17 | 50 | 9 | 58 | | 12 | 55 |
|  |  | 45.70% | | 36.17% | 36.20% | 45.50% | 46.20% | 33.30% | 38.70% | 37.00% | 38.50% | 25.00% | 41.40% | | 37.50% | 38.20% |
|  |  |  | | p=0.058 |  |  | p=0.065 |  | p=0.011 |  | p=0.170 |  | p=0.128 | |  | p=0.083 |
|  |  | mutation | | wildtype | wildtype | other mutation | V3305_S3320del | mutation | wildtype | mutation | wildtype | high | low | | high | low |
| AJCC_M | M0 | 35 | | 137 | 137 | 22 | 13 | 21 | 151 | 44 | 128 | 35 | 137 | | 31 | 141 |
|  |  | 100.00% | | 97.20% | 97.20% | 100.00% | 100.00% | 100.00% | 97.40% | 95.70% | 98.50% | 97.20% | 97.90% | | 96.90% | 97.90% |
|  | M1 | 0 | | 4 | 4 | 0 | 0 | 0 | 4 | 2 | 2 | 1 | 3 | | 1 | 3 |
|  |  | 0.00% | | 2.80% | 2.80% | 0.00% | 0.00% | 0.00% | 2.60% | 4.30% | 1.50% | 2.80% | 2.10% | | 3.10% | 2.10% |
|  |  |  | | p=0.586 |  |  | p=0.407 |  | p=0.456 |  | p=0.280 |  | p=0.820 | |  | p=0.721 |
|  |  | mutation | | wildtype | wildtype | other mutation | V3305_S3320del | mutation | wildtype | mutation | wildtype | high | low | | high | low |
| AJCC_Stage | Stage I | 13 | | 19 | 19 | 9 | 4 | 9 | 23 | 18 | 14 | 17 | 15 | | 15 | 17 |
|  |  | 37.10% | | 13.50% | 13.50% | 40.90% | 30.80% | 42.90% | 14.80% | 39.10% | 10.80% | 47.20% | 10.70% | | 46.90% | 11.80% |
|  | Stage II | 13 | | 43 | 43 | 10 | 3 | 7 | 49 | 12 | 44 | 11 | 45 | | 9 | 47 |
|  |  | 37.10% | | 30.50% | 30.50% | 45.50% | 23.10% | 33.30% | 31.60% | 26.10% | 33.80% | 30.60% | 32.10% | | 28.10% | 32.60% |
|  | Stage III | 9 | | 75 | 75 | 3 | 6 | 5 | 79 | 14 | 70 | 7 | 77 | | 7 | 77 |
|  |  | 25.70% | | 53.20% | 53.20% | 13.60% | 46.20% | 23.80% | 51.00% | 30.40% | 53.80% | 19.40% | 55.00% | | 21.90% | 53.50% |
|  | Stage IV | 0 | | 4 | 4 | 0 | 0 | 0 | 4 | 2 | 2 | 1 | 3 | | 1 | 3 |
|  |  | 0.00% | | 2.80% | 2.80% | 0.00% | 0.00% | 0.00% | 2.60% | 4.30% | 1.50% | 2.80% | 2.10% | | 3.10% | 2.10% |
|  |  |  | | p=0.002 |  |  | p=0.004 |  | p=0.014 |  | p<0.001 |  | p<0.001 | |  | p<0.001 |
|  |  | mutation | | wildtype | wildtype | other mutation | V3305_S3320del | mutation | wildtype | mutation | wildtype | high | low | | high | low |
| Histology | Adenocarcinoma | 33 | | 113 | 113 | 20 | 13 | 19 | 127 | 40 | 106 | 33 | 113 | | 30 | 116 |
|  |  | 94.30% | | 90.40% | 90.40% | 90.90% | 100.00% | 95.00% | 90.70% | 93.00% | 90.60% | 97.10% | 89.70% | | 96.80% | 89.90% |
|  | Mucious | 0 | | 8 | 8 | 0 | 0 | 0 | 8 | 1 | 7 | 0 | 8 | | 0 | 8 |
|  |  | 0.00% | | 6.40% | 6.40% | 0.00% | 0.00% | 0.00% | 5.70% | 2.30% | 6.00% | 0.00% | 6.30% | | 0.00% | 6.20% |
|  | Signet ring cell | 2 | | 4 | 4 | 2 | 0 | 1 | 5 | 2 | 4 | 1 | 5 | | 1 | 5 |
|  |  | 5.70% | | 3.20% | 3.20% | 9.10% | 0.00% | 5.00% | 3.60% | 4.70% | 3.40% | 2.90% | 4.00% | | 3.20% | 3.90% |
|  |  |  | | p=0.110 |  |  | p=0.175 |  | p=0.323 |  | p=0.570 |  | p=0.131 | |  | p=0.165 |
|  |  | mutation | | wildtype | wildtype | other mutation | V3305_S3320del | mutation | wildtype | mutation | wildtype | high | low | | high | low |
| Grade | G1 well differentiated | 2 | | 3 | 3 | 1 | 1 | 0 | 5 | 1 | 4 | 2 | 3 | | 1 | 4 |
|  |  | 6.70% | | 2.40% | 2.40% | 5.30% | 9.10% | 0.00% | 3.60% | 2.40% | 3.50% | 6.30% | 2.40% | | 3.40% | 3.20% |
|  | G2 moderately differentiated | 5 | | 29 | 29 | 4 | 1 | 7 | 27 | 9 | 25 | 8 | 26 | | 6 | 28 |
|  |  | 16.70% | | 23.20% | 23.20% | 21.10% | 9.10% | 38.90% | 19.70% | 22.00% | 21.90% | 25.00% | 21.10% | | 20.70% | 22.20% |
|  | G3 poorly differentiated | 23 | | 91 | 91 | 14 | 9 | 11 | 103 | 30 | 84 | 22 | 92 | | 22 | 92 |
|  |  | 76.70% | | 72.80% | 72.80% | 73.70% | 81.80% | 61.10% | 75.20% | 73.20% | 73.70% | 68.80% | 74.80% | | 75.90% | 73.00% |
|  | G4 undifferentiated | 0 | | 2 | 2 | 0 | 0 | 0 | 2 | 1 | 1 | 0 | 2 | | 0 | 2 |
|  |  | 0.00% | | 1.60% | 1.60% | 0.00% | 0.00% | 0.00% | 1.50% | 2.40% | 0.90% | 0.00% | 1.60% | | 0.00% | 1.60% |
|  |  |  | | p=0.468 |  |  | p=0.756 |  | p=0.220 |  | p=0.893 |  | p=0.534 | |  | p=0.829 |
|  |  |  | |  |  |  |  |  |  |  |  |  |  | |  |  |

**Table S8: Youden index of different mutation numbers in SYSUCC cohort.**

| Mutation numbers of MUC4+MUC16+TTN | Youden index |
| --- | --- |
| 0.5 | 0.404 |
| 1.5 | 0.488 |
| 2.5 | 0.464 |
| 3.5 | 0.369 |
| 4.5 | 0.309 |
| 6 | 0.226 |
| 7.5 | 0.167 |
| 8.5 | 0.083 |

**Table S9: Youden index of different mutation numbers in pan-cancer cohort.**

| Mutation numbers | Youden index of MUC4+TTN | Youden index of 3 genes |
| --- | --- | --- |
| 1.5 | 0.385 | 0.5 |
| 2.5 | 0.63 | 0.6 |
| 3.5 | 0.715 | 0.665 |
| 4.5 | 0.785 | 0.73 |
| 5.5 | 0.788 | 0.764 |
| 6.5 | 0.736 | 0.784 |
| 7.5 | 0.751 | 0.783 |
| 8.5 | 0.726 | 0.793 |
| 9.5 | 0.659 | 0.767 |
| 10.5 | 0.602 | 0.751 |
| 11.5 | 0.526 | 0.751 |
| 12.5 | 0.464 | 0.756 |
| 13.5 | 0.444 | 0.725 |
| 14.5 | 0.424 | 0.684 |
| 15.5 | 0.362 | 0.623 |
| 16.5 | 0.322 | 0.582 |
| 17.5 | 0.281 | 0.505 |

**Table S10: Gene mutation status and mutation numbers in predicting clinical response to immunotherapy in SYSUCC cohort.**

|  | | Clinical Status | | Total | Fisher Precise Test P value |
| --- | --- | --- | --- | --- | --- |
|  |  | Non-Response | Response |  |  |
| MUC4 status | mutation | 3 | 1 | 4 | P=0.436 |
|  | wildtype | 44 | 6 | 50 |  |
| MUC16 status | mutation | 11 | 4 | 15 | P=0.084 |
|  | wildtype | 36 | 3 | 39 |  |
| TTN status | mutation | 20 | 4 | 24 | P=0.687 |
|  | wildtype | 27 | 3 | 30 |  |
| Mutation numbers of MUC4+MUC16+TTN | high | 8 | 4 | 12 | P=0.036 |
|  | low | 39 | 3 | 42 |  |
| Total | | 47 | 7 | 54 |  |

**Table S11: Gene mutation status, mutation numbers and TMB in predicting clinical response to immunotherapy in pan-cancer cohort.**

|  | | Clinical Status | | Total | Fisher Precise Test P value |
| --- | --- | --- | --- | --- | --- |
|  |  | Non-Response | Response |  |  |
| MUC4 status | mutation | 24 | 13 | 37 | P=0.303 |
|  | wildtype | 155 | 57 | 212 |  |
| MUC16 status | mutation | 100 | 46 | 146 | P=0.156 |
|  | wildtype | 79 | 24 | 103 |  |
| TTN status | mutation | 117 | 52 | 169 | P=0.175 |
|  | wildtype | 62 | 18 | 80 |  |
| Mutation numbers of MUC4+TTN | high | 33 | 15 | 48 | P=0.590 |
|  | low | 146 | 55 | 201 |  |
| Mutation numbers of MUC4+MUC16+TTN | high | 33 | 15 | 48 | P=0.590 |
|  | low | 146 | 55 | 201 |  |
| TMB | high | 30 | 19 | 49 | P=0.064 |
|  | low | 149 | 51 | 200 |  |
| Total | | 179 | 70 | 249 |  |
